# Supplementary material for: Ecological memory preserves phage resistance mechanisms in bacteria
Source: Nat Commun. 2021 Nov 24;12:6817. doi: 10.1038/s41467-021-26609-w (PMC8613279; doi:10.1038/s41467-021-26609-w)
Supplement: Supplementary file 2 — Supplementary Information [file 41467_2021_26609_MOESM2_ESM.pdf]

Ecological memory preserves phage resistance mechanisms in bacteria

Supplementary Information

Antun Skanata* and Edo Kussell†
Center for Genomics and Systems Biology, Department of Biology, New York University, New York, NY 10003.

* askanata@syr.edu; Currently at Department of Physics, Syracuse University, Syracuse, NY 13210.
† edo.kussell@nyu.edu; Also at Department of Physics, New York University, New York, NY 10003.

Supplementary Figures

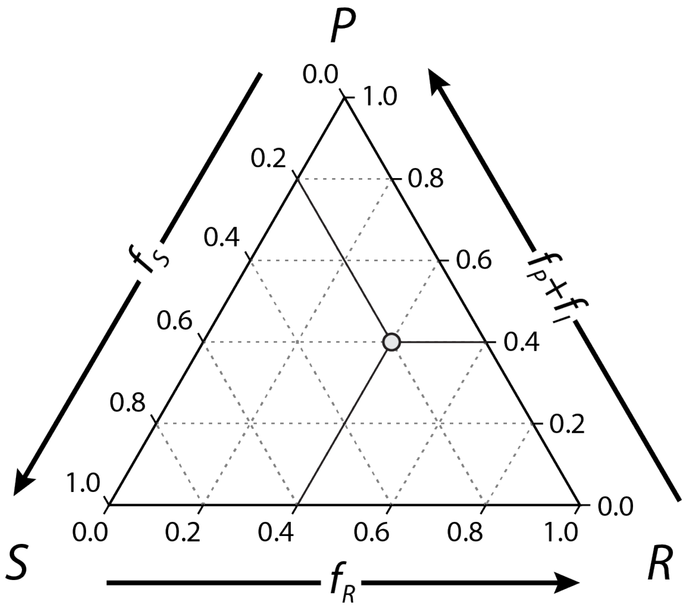

Supplementary Figure 1. A legend showing how to read flow diagrams as triangle plots. Corners correspond to pure populations of sensitive bacteria (S), resistant bacteria (R), or phage (P). Frequencies of resistant, sensitive, and infected host and phage increase in the direction of the arrows along the edges of the simplex. Interior points correspond to mixtures of bacteria and phage, whose frequencies can be read by following dotted lines as illustrated in this example.

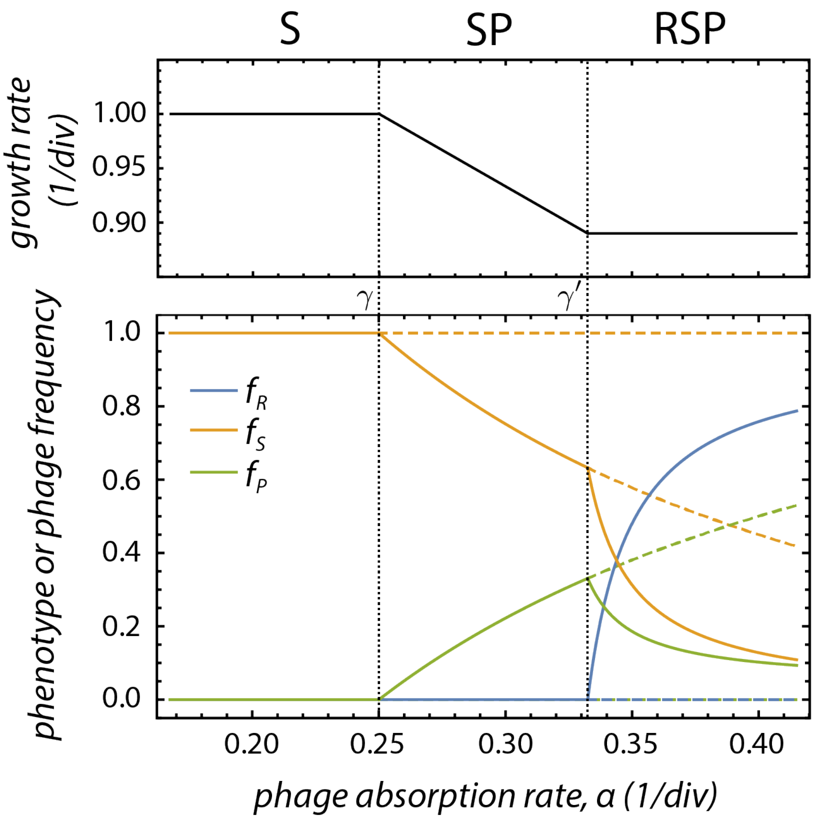

Supplementary Figure 2. Plot showing the growth rate of host cells and the phenotype and phage frequencies in the model of preventative defenses as α is varied across γ and γ' at burst size $\beta = 10$. The system goes through a series of two transcritical bifurcations. S, SP and RSP denote stable phases of phage-host dynamics, corresponding to the fixation of sensitive bacteria (S), coexistence of sensitive bacteria and phage (SP), or coexistence of resistant and sensitive host and phage (RSP). Solid curves - stable fixed points, dashed curves - unstable fixed points. Remaining parameters are $n_{\rm r} = 1$, $d = 1$, $b = 0.9$, $k_{\rm L} = 1$, $s = 0.01$, $K_{\rm m} = 0$. From Eq. 12 we observe that when the RSP fixed point is stable, the growth rate of host cells is $\lambda = b - s$. For stable fixed points with $f_R = 0$, we use $\dot{f}_S = 0$ to obtain $\lambda = d$ in the S phase and $\lambda = (d - \alpha n_{\rm r})(\beta k_{\rm L} - d - k_{\rm L})/(\beta k_{\rm L} - (1 + n_{\rm r})(d + k_{\rm L}))$ in the SP phase. For periodic dynamics in the RSP phase, at steady state the average growth rate can be obtained by integrating $\dot{f}_R/f_R$ over the period T: $\frac{1}{T}(\log f_R(t+T) - \log f_R(t)) = b - s - \langle\lambda\rangle_T$, where $\langle\lambda\rangle_T = \frac{1}{T}\int_t^{t+T} \lambda(t)dt$. Since $f_R(t+T) = f_R(t)$, we obtain $\langle\lambda\rangle_T = b - s$, the growth rate of the resistant phenotype.

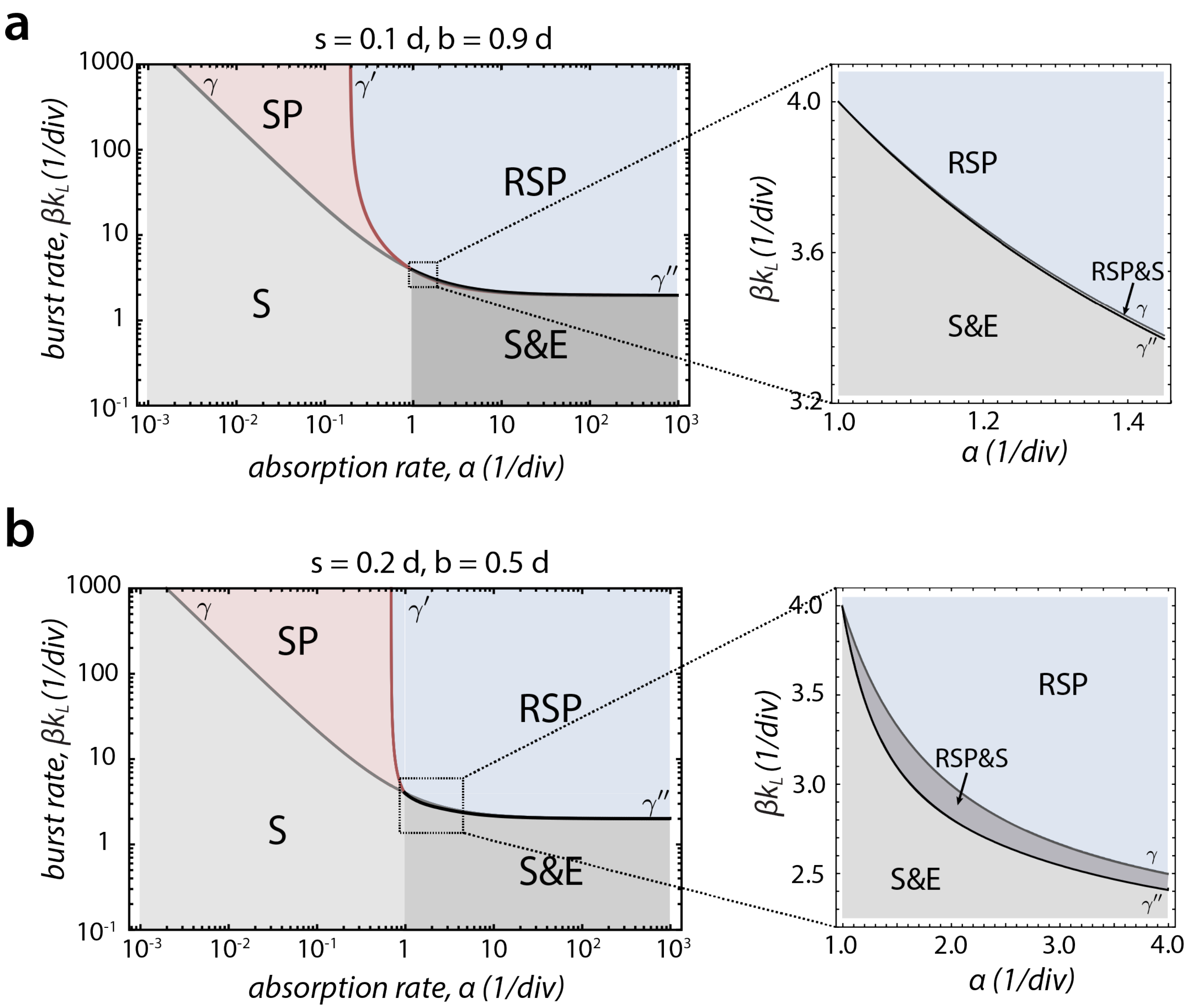

Supplementary Figure 3. Phase diagrams for preventative defenses for larger switching rates (panels a and b) and larger costs of defense mechanism, $d - b$ (panel b). Insets show the separation of γ and γ'' for $\alpha > d$. The RSP&S bistable region located between γ and γ'' curves becomes larger with the increase in switching rate and cost $d - b$. Remaining parameters are $n_{\rm r} = 1$, $d = 1$, $k_{\rm L} = 1$, $K_{\rm m} = 0$.

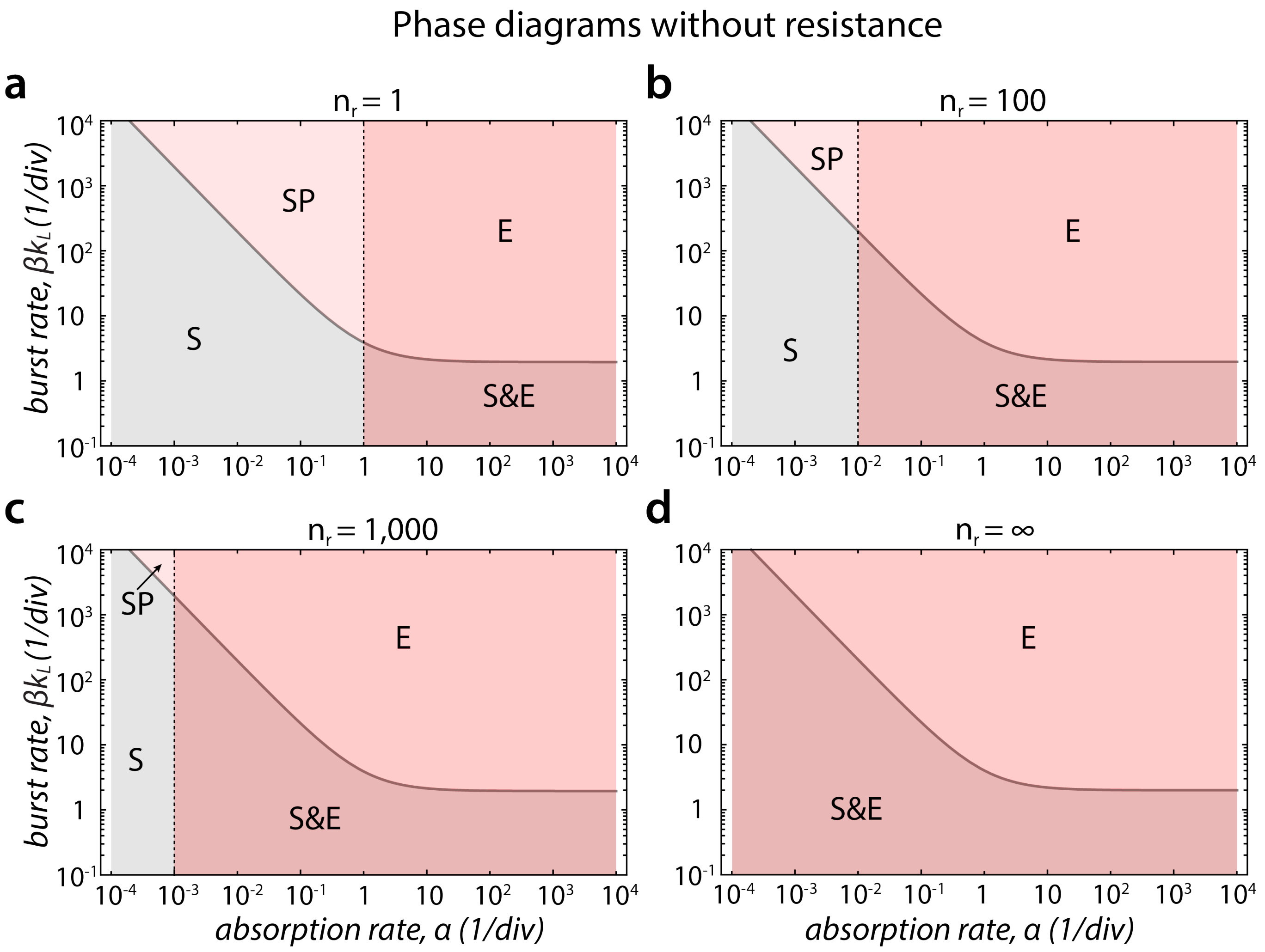

Supplementary Figure 4. Phase diagrams without resistance, as a function of the number of receptors n_{r}, phage burst rate βk_{L} and phage absorption rate α. Panels (a-d) correspond to increasing numbers of receptors on the cells, from $n_{\mathrm{r}} = 1$ (panel a), $n_{\mathrm{r}} = 100$ (panel b), $n_{\mathrm{r}} = 1,000$ (panel c) to the limiting value of $n_{\mathrm{r}} = \infty$ (panel d). The distinct stable outcomes correspond to the S, SP and E phases, as defined in the text. Vertical dotted line indicates $\alpha = d/n_{\mathrm{r}}$. Host extinction is stable for $\alpha > d/n_{\mathrm{r}}$. Parameters used are $d = 1$, $k_{\mathrm{L}} = 1$ and $K_{\mathrm{m}} = 0$.

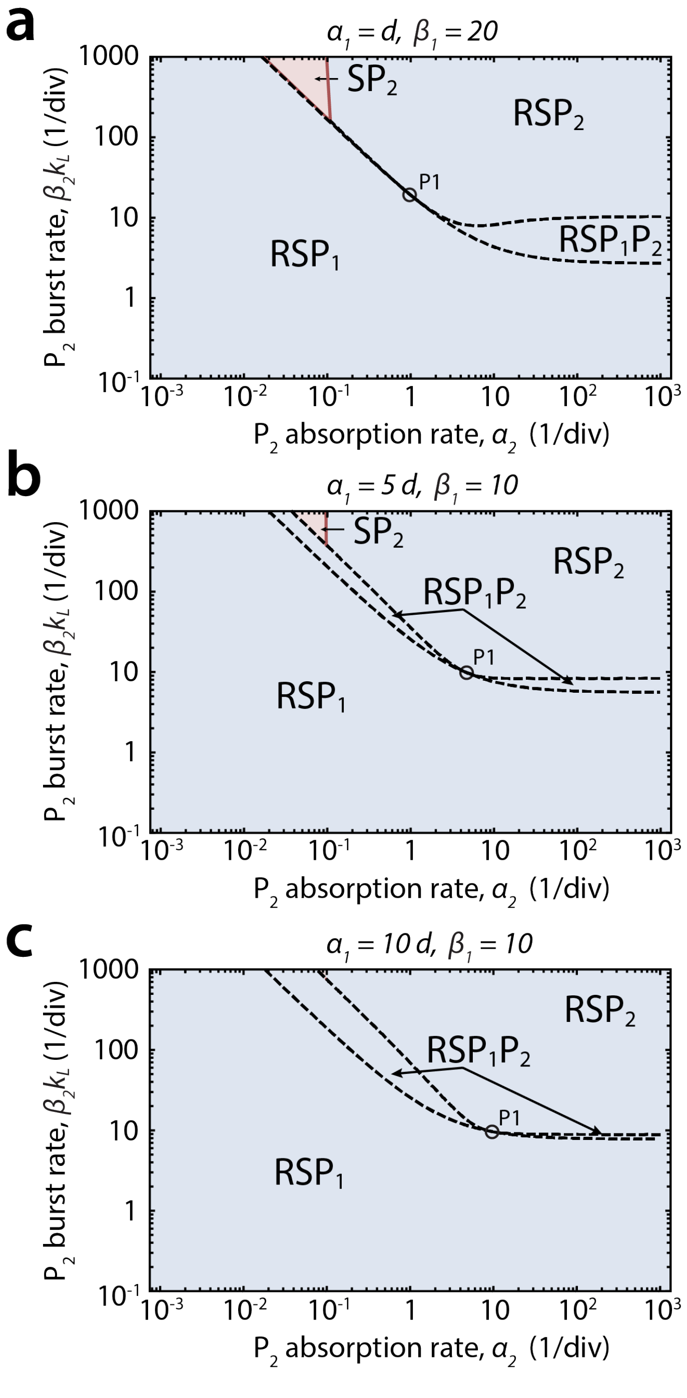

Supplementary Figure 5. Phase diagrams for two phage types, P_1 and P_2, shown for P_2 parameters in the model of preventative defenses. (a-c) P_1 parameters (α_1, β_1) are located in the RSP phase. Dashed curves separate regions of single- and multi-phage phases. The pink region corresponds to the case where P_2 drives P_1 and the resistant phenotype extinct. The resistant phenotype switches at a rate $s = 10^{-3}$. Size of the coexistence regions weakly depends on s. Remaining parameters are $d = 1$, $b = 0.9$, $k_{\mathrm{L}} = 1$, $K_{\mathrm{m}} = 0$.

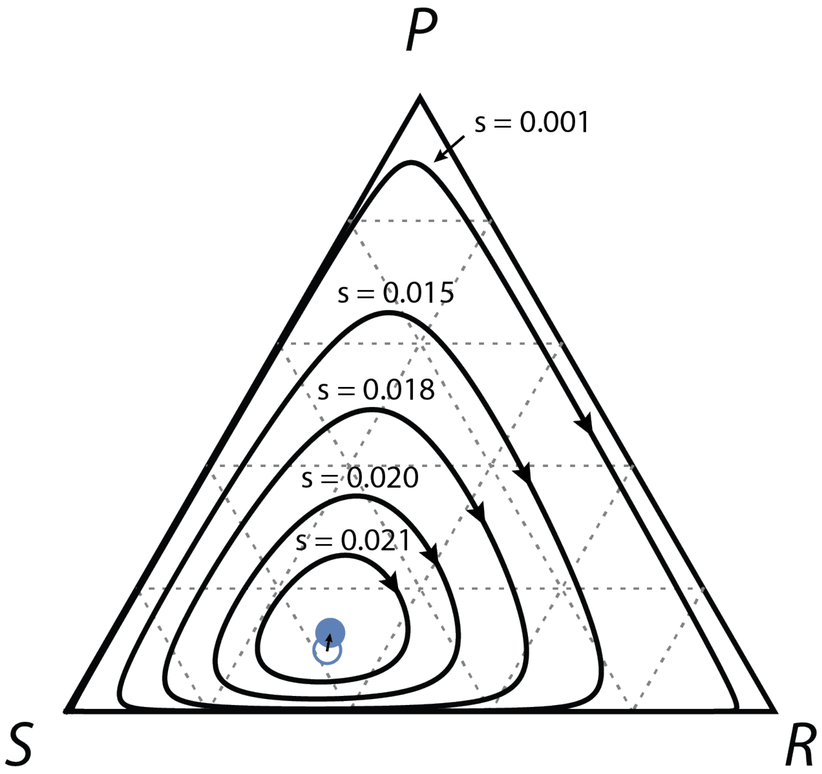

Supplementary Figure 6. Ternary plot of limit cycle periodic orbits in the model of immune defenses for $\alpha = 1.5$, $\beta = 5$ and s varied from 0.001 to 0.021. The unstable interior fixed point (empty blue circle) drifts with the increase of s in the direction of the arrow, and becomes stable for $s \approx 0.0216$, which is represented with the filled circle. The periodic trajectories contract into the interior of the simplex and around the fixed point. Remaining parameters are $n_{\mathrm{r}} = 1$, $d = 1$, $b = 0.9$, $k_{\mathrm{L}} = 1$, $K_{\mathrm{m}} = 0$.

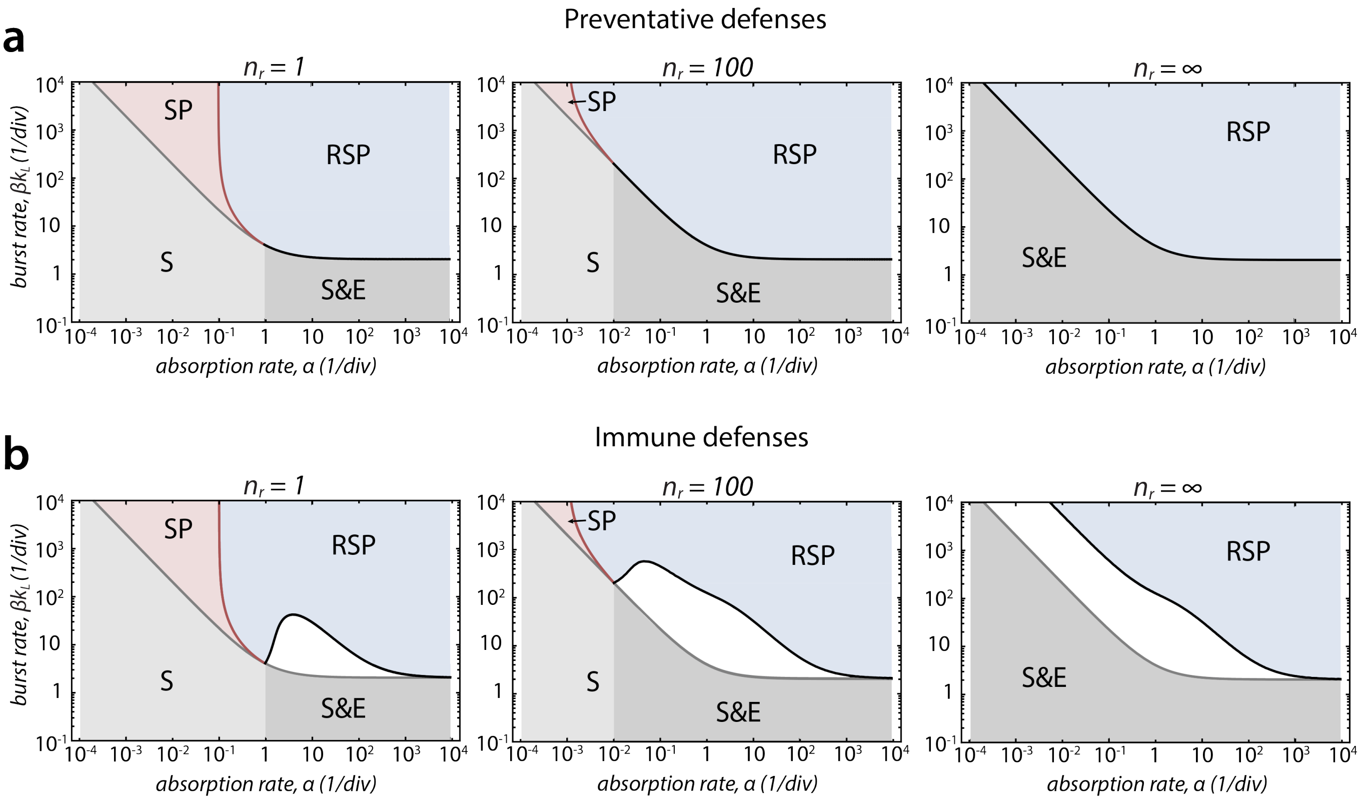

Supplementary Figure 7. Phase diagrams for (a) preventative and (b) immune defenses, for different values of n_r. We consider the phase structure of Eq. (12) for a range of n_r including $n_\mathrm{r} \gg 1$. In the limit $n_\mathrm{r} \to \infty$ the dependence on n_r drops out, as $\lim_{n_\mathrm{r}\to\infty} k_\mathrm{I} = \alpha P/A$. As n_r increases the SP phase moves to lower values of $\alpha < d/n_\mathrm{r}$ while the RSP phase for immune defenses develops a larger region where periodic dynamics are possible (shown in white). In the limit $n_\mathrm{r} \to \infty$ the SP phase disappears and the phase diagram contains only the S and RSP phases separated by $\gamma = (d+\alpha)(d+k_\mathrm{L})/\alpha$. Parameters used to generate the figure are $d = 1$, $b = 0.9$, $s = 10^{-3}$, $K_\mathrm{m} = 0$.

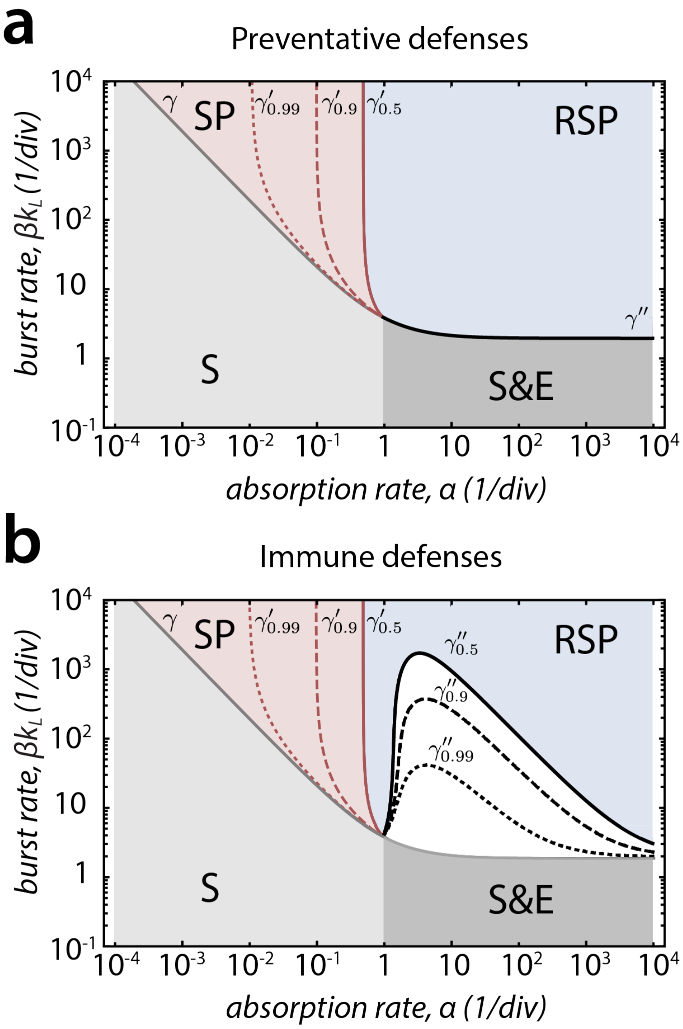

Supplementary Figure 8. Phase diagrams for (a) preventative and (b) immune defenses, for different values of b ($b = 0.5$ - solid, $b = 0.9$ - dashed, $b = 0.99$ - dotted), corresponding to different fitness costs of defense. Bifurcation curves γ' and γ'' are labelled according to their respective b values. For preventative defenses changes in γ'' occur within the width of the curve and feature a narrow RSP&S bistable region at the location of γ'' curve. The location of γ does not depend on b. Remaining parameters are $n_{\rm r} = 1$, $d = 1$, $s = 10^{-4}$, $K_{\rm m} = 0$.

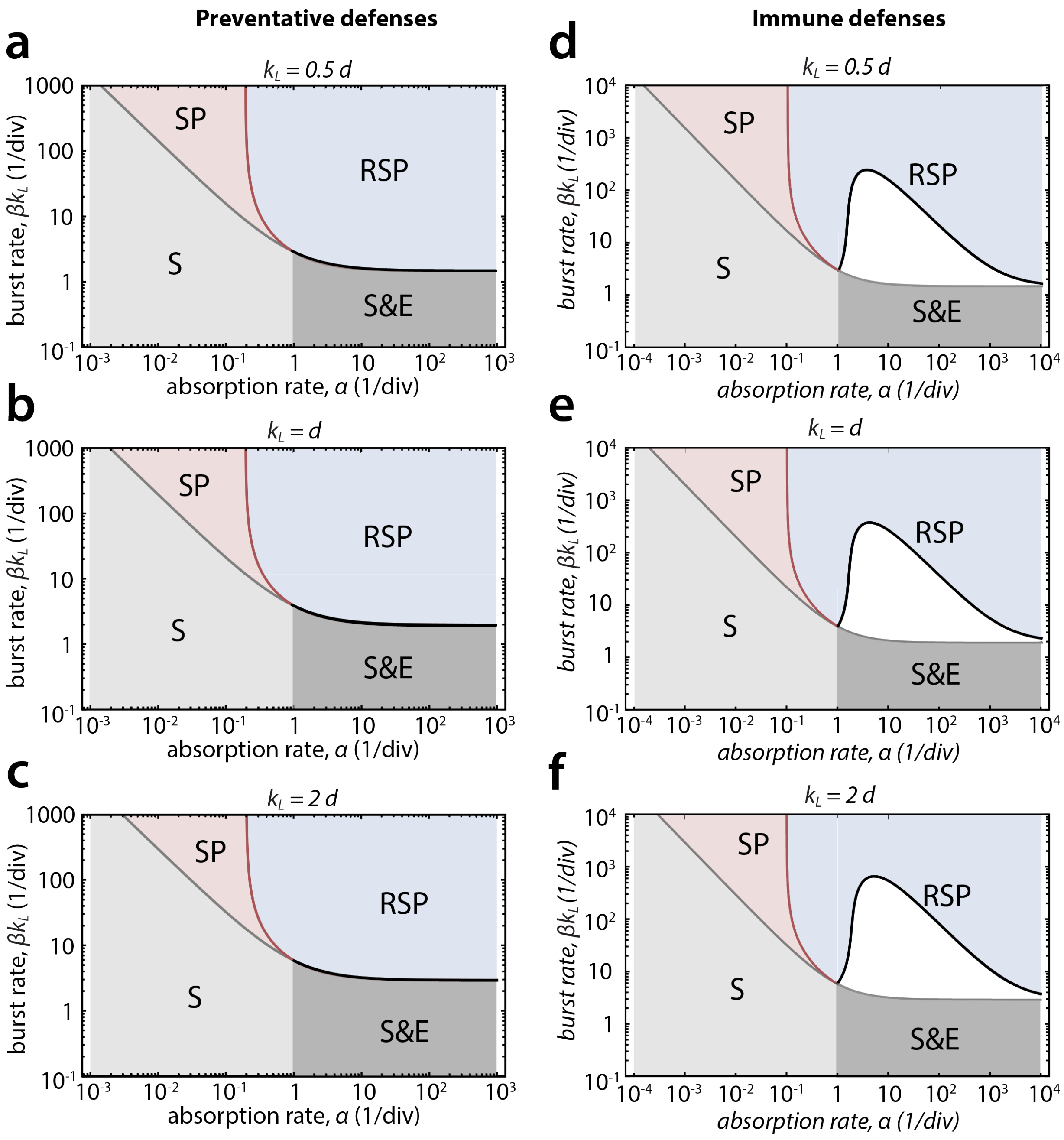

Supplementary Figure 9. Phase diagrams for (a-c) preventative and (d-f) immune defenses , for different values of k_{L}. We varied k_{L} by a factor of two above and below cell division rate d to show that the phase structure remains unchanged over the range that is commonly found in experiments. Larger deviations of k_{L} similarly do not affect the phase structure. Remaining parameters are $n_{\mathrm{r}} = 1$, $d = 1$, $b = 0.9$, $s = 10^{-4}$, $K_{\mathrm{m}} = 0$.

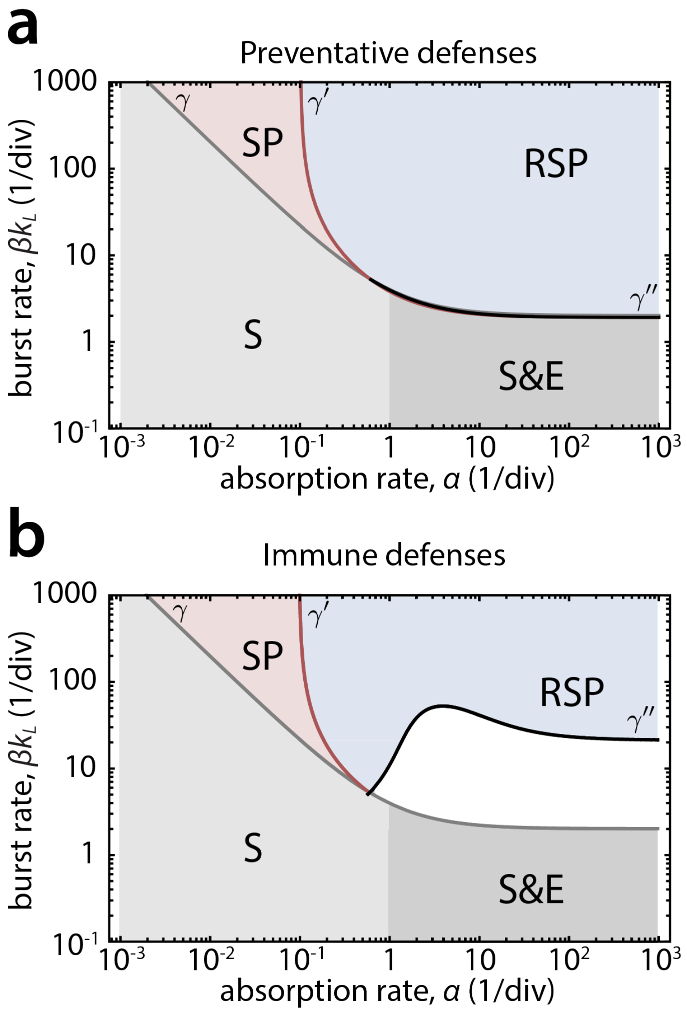

Supplementary Figure 10. Phase diagrams for (a) preventative and (b) immune defenses where the infected phenotype does not absorb phage. Here we consider phage that does not fall off the receptor upon infection and therefore blocks the receptor for subsequent absorption of phage in the infected phenotype. This effect would be small for large n_{r} and most extreme in the model of minimal sensitivity ($n_{\mathrm{r}} = 1$), which would prevent the infected phenotype to act as a phage sink. We solved the $n_{\mathrm{r}} = 1$ model to find this change shifts the location of γ' and γ'' bifurcations as well as impacts the dynamics at the intersection of S, SP and RSP phases. Parameters used are $n_{\mathrm{r}} = 1$, $d = 1$, $b = 0.9$, $k_{\mathrm{L}} = 1$, $s = 10^{-3}$, $\kappa' = 0$.

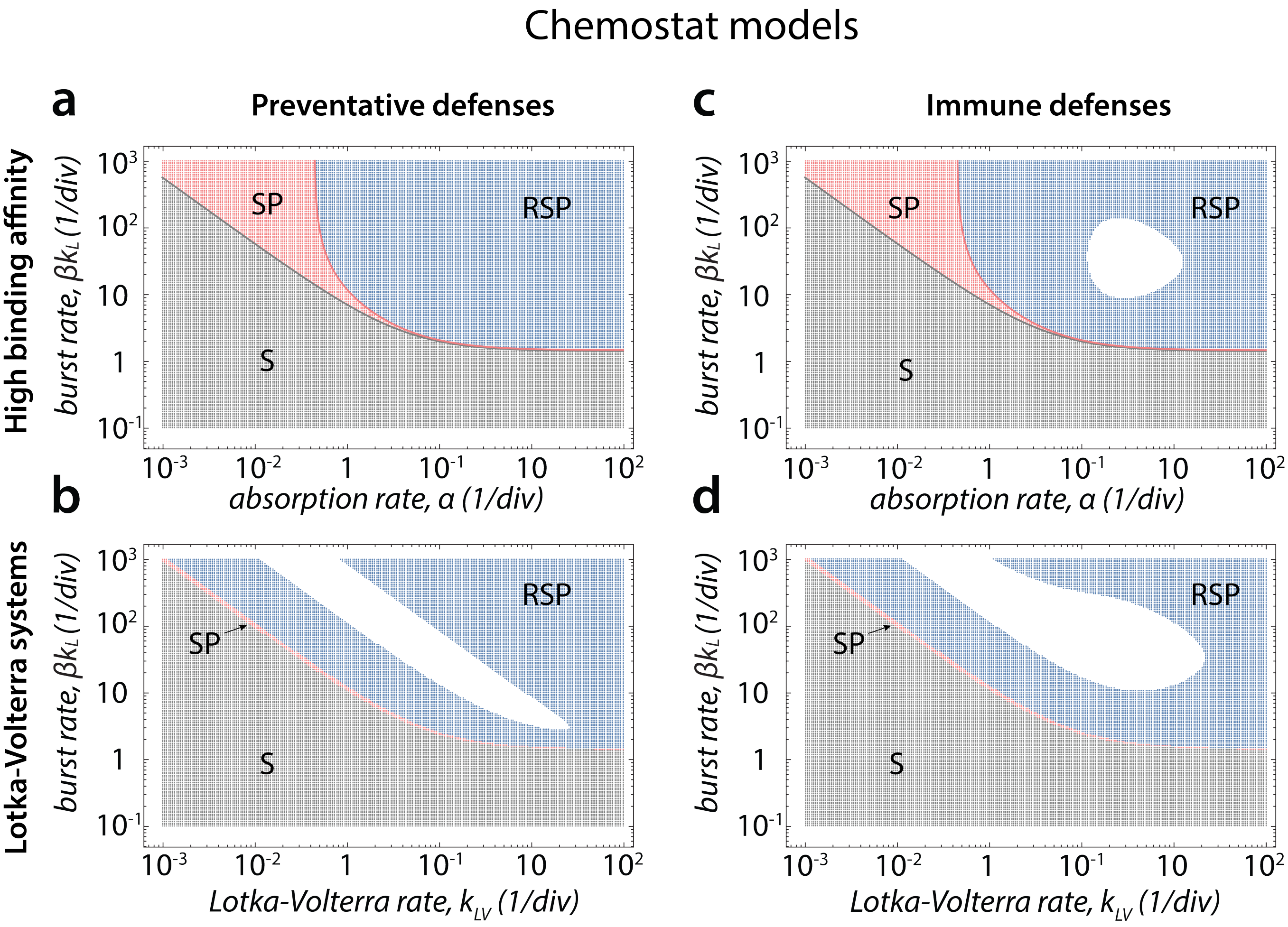

Supplementary Figure 11. Phases of stable fixed points in chemostat models, numerically obtained. (a-b) preventative defenses in the high binding affinity limit (infection rate of the form $k_{\rm I} = \alpha P/(A + P)$) and the Lotka-Volterra limit ($k_{\rm I} = k_{LV} P$), (c-d) immune defenses in the high binding affinity and the Lotka-Volterra limits. White regions indicate the absence of stable fixed points. Parameters used: $n_{\rm r} = 1$, $d = 1$, $b = 0.9$, $k_{\rm L} = 1$, $D = 0.4$, $\rho_{in} = 1$, $s = 10^{-4}$.

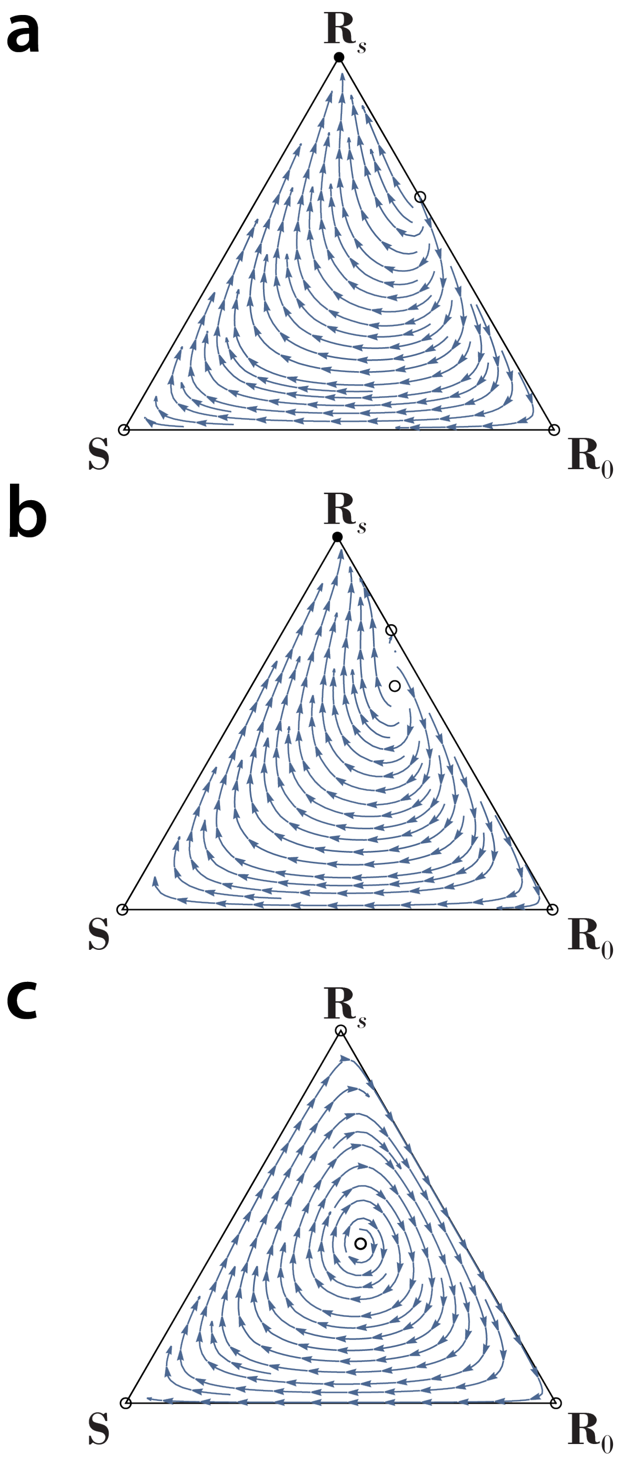

Supplementary Figure 12. Flow diagrams of the replicator equation for the payoff matrix given with Supplementary Eq. (8) in the limit $g_1, g_2 \gg c$, for (a) $s = c/4$ ($a_3 = 2/5$), (b) $s = c/2$ ($a_3 = 2/3$), and (c) $s = 2c$ ($a_3 = 4/3$). Stable (unstable) fixed points are shown as solid (open) circles.

Supplementary Tables

Strategy	Fixed point?	Nash Eq?	ESS?
$\mathbf{R}_s$	Yes	$a_3 \leq 1$	$a_3 < 1$
$\mathbf{R}_0$	Yes	No	No
$\mathbf{S}$	Yes	No	No
$\mathbf{y}_1 \equiv \frac{1}{2-a_3}\mathbf{R}_s + \frac{1-a_3}{2-a_3}\mathbf{R}_0$	$a_3 < 1$	$\frac{a_2-1}{a_2} \leq a_3 < 1$	No
$\mathbf{y}_2 \equiv r_1\mathbf{R}_s + r_2\mathbf{R}_0 + r_3\mathbf{S}$	$a_3 > \frac{a_2-1}{a_2}$	$a_3 > \frac{a_2-1}{a_2}$	No

Supplementary Table I. Fixed points of the replicator equation for payoff matrix given in Supplementary Eq. (8). Each listed strategy is a fixed point when the given conditions are satisfied, with additional conditions for Nash equilibria or ESS. The coefficients r_i are given by $r_1 \equiv [1 + a_1(a_2 - 1)]/Q$,$r_2 \equiv [1 + a_3(a_1 - 1)]/Q$, $r_3 \equiv [1 + a_2(a_3 - 1)]/Q$, where $Q \equiv 3 + a_1a_2 + a_1a_3 + a_2a_3 - a_1 - a_2 - a_3$.

Supplementary Notes

Supplementary Note 1. MODELING TURBIDOSTAT AND CHEMOSTAT CONTROL MECHANISMS

A. Turbidostat control of host biomass

We consider the control of host biomass by a feedback dilution mechanism that maintains a fixed host concentration within the system volume. The dynamics are given by transforming the simplex equations (12) to a set of coordinates given by phenotype frequencies in the host population $x = R/(R+S+I)$, $y = S/(R+S+I)$, $z = I/(R+S+I) = 1-x-y$ and phage pressure $p = P/(R+S+I)$. In this basis, the infection rate is $k_{\mathrm{I}} = \alpha n_{\mathrm{r}} p/(K''_{\mathrm{m}} + n_{\mathrm{r}} a + p)$, with $K''_{\mathrm{m}} \equiv K_{\mathrm{m}}/(R+S+I)$ and where $a = A/(R+S+I)$ is total frequency of phage-adsorbing hosts, which equals $1-x$ for preventative and 1 for immune defenses. The turbidostat equations are:

$$
\begin{aligned}
\dot{x} &= (b-s)x - \lambda(t)x, \\
\dot{y} &= dy + sx - k_{\mathrm{I}}y - \lambda(t)y, \\
\dot{z} &= k_{\mathrm{I}}y - k_{\mathrm{L}}z - \lambda(t)z, \\
\dot{p} &= -k_{\mathrm{I}}a + \beta k_{\mathrm{L}}z - \lambda(t)p
\end{aligned}
\tag{1}
$$

where $\lambda(t) = dy + bx - k_{\mathrm{L}}z$ is the instantaneous host growth rate. These coordinates can be mapped to the simplex via:

$$
\begin{aligned}
f_R &\leftrightarrow \frac{x}{1+\nu p} \qquad f_S \leftrightarrow \frac{y}{1+\nu p} \\
f_I &\leftrightarrow \frac{z}{1+\nu p} \qquad f_P \leftrightarrow \frac{\nu p}{1+\nu p}
\end{aligned}
\tag{2}
$$

except at the host extinction point E, which is a singular outcome in this basis and needs to be studied on the simplex. The stability of fixed points in the host biomass basis matches that on the simplex and generates the same phases as the ones presented for the simplex equations.

B. Chemostat control of host biomass

We analyze the dynamics in a chemostat model given by the equations

$$
\begin{aligned}
\dot{\rho}(t) &= -b(\rho)\eta R(t) - d(\rho)\eta S(t) + D[\rho_{in} - \rho(t)], \\
\dot{R}(t) &= b(\rho)R(t) - sR(t) - DR(t), \\
\dot{S}(t) &= d(\rho)S(t) + sR(t) - k_{\mathrm{I}}(t)S(t) - DS(t), \\
\dot{I}(t) &= k_{\mathrm{I}}(t)S(t) - k_{\mathrm{L}}I(t) - DI(t), \\
\dot{P}(t) &= -k_{\mathrm{I}}(t)A(t) + \beta k_{\mathrm{L}}I(t) - DP(t)
\end{aligned}
\tag{3}
$$

Here ρ is the resource, η is the resource to growth conversion factor (which we set to 1), ρ_{in} is the concentration of the supplied resource while D is the chemostat dilution rate. R, S, I and P denote concentrations of host and phage, and the infection rate is $k_{\mathrm{I}} = \alpha n_{\mathrm{r}} P/(K_{\mathrm{m}} + n_{\mathrm{r}} A + P)$. Initially, we consider the high binding affinity limit and set $K_{\mathrm{m}} = 0$. Cellular division rates depend on resource concentration and are generally chosen to be Monod functions of the form $d(\rho) = d\,\rho/(\rho_c + \rho)$, where ρ_c is nutrient concentration at half-maximal growth rate. We consider the regime of nutrient limitation $\rho \ll \rho_c$, for which division rates linearly depend on ρ: $d(\rho) = d \cdot \rho/\rho_c$, and similarly $b(\rho) = b \cdot \rho/\rho_c$; we absorb ρ_c into the prefactor by setting $\rho_c = 1$. Extinction of both phage and host is globally stable if $D > d \cdot \rho_{in}$, which means the population is washed out faster than it can grow. Dilution rates below $d \cdot \rho_{in}$ but above $b \cdot r_{in}$ will select against the resistant phenotype. We will consider dilution rates $D < b \cdot \rho_{in} - s$ so that the chemostat supports growth of all phenotypes.

The analysis of chemostat fixed points gives the same phases as in the turbidostat formulation with: (i) the E phase, which is unstable for our choice of dilution rate; (ii) the S phase, corresponding to the solution $\rho = D/d$, $S = \rho_{in} - \rho$, $R = L = P = 0$, which is stable for

$$
\beta k_{\mathrm{L}} < \gamma_C \equiv \frac{(D+\alpha)(D+k_{\mathrm{L}})}{\alpha};
\tag{4}
$$

(iii) the SP phase which is stable for $\gamma_C < \beta k_{\rm L} < \gamma'_C$ where

$$\gamma'_C \equiv \frac{(d(D+s)+bk_{\rm L})(bn_{\rm r}(D+\alpha)-D(d-b)-ds)}{b(bn_{\rm r}\alpha - D(d-b)-ds)}; \tag{5}$$

and (iv) the RSP phase, whose stability we determine numerically. Since the dilution rate controls the overall growth rate and therefore the relative fitness differences between phenotypes, changing the dilution rate will impact the stability of the fixed points and can lead to removal or enhancement of regions that admit periodic dynamics in the phase diagram.

Supplementary Figure 11 shows the representative phase diagrams of stable fixed points in the chemostat model for preventative and immune defenses, and in the low binding affinity or Lotka-Volterra limit where the infection rates are $k_{\rm I} = k_{LV}P$ with $k_{LV} = \alpha n_{\rm r}/K_{\rm m}$.

Supplementary Note 2. PHASE DIAGRAMS WITHOUT RESISTANCE

We consider the phage-bacteria system given by Eq. (12) in the main text, and set $f_R = 0$ to model the system where the host has not acquired resistance to phage. Supplementary Fig. 4 shows the phase diagrams displaying the regions of stable S, SP and E fixed points in the absence of resistant phenotype. The eigenvalues at the host extinction fixed point $\{f_S, f_I\} = \{0, 0\}$ are

$$\begin{aligned} L_1^E &= d - n_{\rm r}\alpha, \\ L_2^E &= -k_{\rm L}, \end{aligned} \tag{6}$$

which make extinction stable for $\alpha > d/n_{\rm r}$. The S phase $\{f_S, f_I\} = \{1, 0\}$ is stable for $\beta k_{\rm L} < \gamma$. Thus, there is a bistable region at $\alpha > d/n_{\rm r}$ and $\beta k_{\rm L} < \gamma$ which is shown in Supplementary Fig. 4. The SP phase is stable for $\alpha < d/n_{\rm r}$ and $\beta k_{\rm L} > \gamma$. In this reduced model, in the region where E is stable there can exist a second extinction fixed point, such that $f_S = 0$ and $f_I = \text{const} > 0$, and in which infected host and phage decay exponentially. This pathological fixed point is never stable in the full model with resistance, but in the reduced model we consider it part of the E phases.

Supplementary Note 3. SWITCHING MAINTAINS MULTIPLE PHAGE TYPES IN THE PREVENTATIVE DEFENSE MODEL

We consider two phage types P_1 and P_2 that compete for the same host. In this model, a host cannot be simultaneously infected by more than one phage type, but the infected cell can absorb phage of any type. The two phage types can have different absorption rates α and burst sizes β. The equations are

$$\begin{aligned} \dot{R} &= (b-s)R - \lambda(t)R, \\ \dot{S} &= dS + sR - \frac{\alpha_1 P_1 + \alpha_2 P_2}{A + P_1 + P_2}S - \lambda(t)S, \\ \dot{I}_1 &= \frac{\alpha_1 P_1}{A + P_1 + P_2}S - k_{\rm L} I_1 - \lambda(t) I_1, \\ \dot{I}_2 &= \frac{\alpha_2 P_2}{A + P_1 + P_2}S - k_{\rm L} I_2 - \lambda(t) I_2, \\ \dot{P}_1 &= -\frac{\alpha_1 P_1}{A + P_1 + P_2}A + \beta_1 k_{\rm L} I_1 - \lambda(t) P_1, \\ \dot{P}_2 &= -\frac{\alpha_2 P_2}{A + P_1 + P_2}A + \beta_2 k_{\rm L} I_2 - \lambda(t) P_2 \end{aligned} \tag{7}$$

where $A = S + I_1 + I_2$. We consider the minimal sensitivity condition $n_{\rm r} = 1$, but the model is easily generalizable for any $n_{\rm r} > 1$. Supplementary Fig. 5 shows the phase diagrams for the two-phage system, which include the RSP$_1$, RSP$_2$, and RSP$_1$P$_2$ phases, where both phages stably coexist. The phase diagram is shown for P_2 given P_1 with coordinates $\{\alpha_1, \beta_1\}$ at three different locations in the RSP phase. For zero switching rate there will be no phage coexistence, since the RSP phase collapses to pure R, which does not support phage growth. Dashed curves denote transitions from single to multiple phage phases where both phage types are maintained in the RSP phase. In the

region where P_2 outcompetes P_1 resistance can be lost if P_2 parameters are located in P_2's SP phase; this region occurs at high P_2 burst rates and is shown in pink. The size and shape of these regions does not strongly depend on the value of $s > 0$.

Supplementary Note 4. FIXED POINT ANALYSIS FOR PATCH INVASION DYNAMICS

We analyze the replicator dynamics for the payoff matrix ϕ given in the main text, which we rewrite as

$$\begin{array}{c} \\ \mathbf{R}_s \\ \mathbf{R}_0 \\ \mathbf{S} \end{array} \begin{array}{c} \mathbf{R}_s\ \mathbf{R}_0\ \mathbf{S} \\ \begin{pmatrix} 1 & 0 & a_1 \\ a_3 & 1 & 0 \\ 0 & a_2 & 1 \end{pmatrix} \end{array} \tag{8}$$

where $a_1 \equiv 2g_1/(g_1 + c)$, $a_2 \equiv 2g_2/(g_2 + c)$, and $a_3 \equiv 2s/(s + c)$. We always have $0 \leq a_i < 2$, and additionally we assume a_1,$a_2 > 1$.

Supplementary Table I lists the possible fixed points, which include the three pure strategies and two mixed strategies, $\mathbf{y}_1$ and $\mathbf{y}_2$. $\mathbf{y}_1$ is a fixed point on the $\mathbf{R}_s$-$\mathbf{R}_0$ edge for $a_3 < 1$ (i.e. $s < c$), and a Nash equilibrium if $\mathbf{S}\phi\mathbf{y}_1 \leq \mathbf{y}_1\phi\mathbf{y}_1$ which occurs for $a_3 \geq 1-1/a_2$. It is not an ESS because $\mathbf{y}_1$ does not beat alternative best replies (e.g. $\mathbf{R}_0\phi\mathbf{y}_1 = \mathbf{y}_1\phi\mathbf{y}_1$, yet $\mathbf{y}_1\phi\mathbf{R}_0 = (1 - a_3)/(2 - a_3) < 1 = \mathbf{R}_0\phi\mathbf{R}_0$). $\mathbf{y}_2$ is a Nash equilibrium in the simplex interior, hence each of the pure strategies is an alternative best reply. For $\mathbf{y}_2$ to be an ESS, it must beat each of the pure strategies, i.e. $\mathbf{y}_2\phi\mathbf{x} > \mathbf{x}\phi\mathbf{x}$ must hold for $\mathbf{x} = \mathbf{R}_s$, $\mathbf{R}_0$, and $\mathbf{S}$. This implies $a_1 - 1 > r_2/r_1$, $a_3 - 1 > r_3/r_2$, and $a_2 - 1 > r_1/r_3$. Multiplying the inequalities we obtain $(a_1-1)(a_2-1)(a_3-1) > 1$ which cannot hold since $0 \leq a_i < 2$, hence $\mathbf{y}_2$ is not an ESS.

For $a_3 \geq 1$ ($s \geq c$), there are no stable fixed points, the interior fixed point $\mathbf{y}_2$ is an unstable focus, and flows in the interior approach the heteroclinic cycle on the boundary. For decreasing a_3, $\mathbf{R}_s$ becomes a Nash equilibrium at $a_3 = 1$ ($s = c$), and then bifurcates to yield the Nash equilibrium $\mathbf{y}_1$ and the unique ESS $\mathbf{R}_s$. As a_3 decreases further, $\mathbf{y}_2$ moves toward the $\mathbf{R}_s$-$\mathbf{R}_0$ edge, and at $a_3 = (a_2 - 1)/a_2$ it merges with $\mathbf{y}_1$ and the latter loses its Nash equilibrium status. At each bifurcation a qualitative change occurs in the flow structure (see Supplementary Fig. 12 for representative plots).

Supplementary Note 5. INDEPENDENT DISPERSION OF PHAGE

To model phage that can migrate independently of bacteria, we add the strategy $\mathbf{P}$ to the game, which corresponds to phage without host cells. Co-invasion events can then involve $\mathbf{P}$ and one of the three bacterial strains $\mathbf{R}_s$, $\mathbf{R}_0$, or $\mathbf{S}$. The game $\mathbf{R}_s$ vs. $\mathbf{P}$ resolves immediately with $\mathbf{R}_s$, as phage is already present with the $\mathbf{R}_s$ strain. Likewise, $\mathbf{R}_0$ vs. $\mathbf{P}$ resolves in favor of $\mathbf{R}_0$ because phage cannot grow on resistant cells and therefore cannot establish. Lastly, in $\mathbf{S}$ vs. $\mathbf{P}$, phage can grow on sensitive cells, and drives $\mathbf{S}$ to extinction. We assign a'_1 as the payoff to $\mathbf{P}$ against $\mathbf{S}$, and note that $\mathbf{R}_s$ outcompetes $\mathbf{S}$ by the same mechanism, i.e. phage-mediated killing of sensitive cells. Since $\mathbf{R}_s$ additionally contains exponentially growing resistant cells, we have $a_1 \geq a'_1$. The patch invasion game dynamics thus have the payoff matrix

$$\begin{array}{c} \\ \mathbf{R}_s \\ \mathbf{R}_0 \\ \mathbf{S} \\ \mathbf{P} \end{array} \begin{array}{c} \mathbf{R}_s\ \mathbf{R}_0\ \mathbf{S}\ \mathbf{P} \\ \begin{pmatrix} 1 & 0 & a_1 & 2 \\ a_3 & 1 & 0 & 2 \\ 0 & a_2 & 1 & 0 \\ 0 & 0 & a'_1 & 1 \end{pmatrix} \end{array} \tag{9}$$

If $a'_1 < a_1$, $\mathbf{P}$ is a dominated strategy and is therefore eliminated, reducing the 4-strategy game given with Supplementary Eq. (9) to the $\mathbf{R}_s\mathbf{R}_0\mathbf{S}$ face discussed above. For $a'_1 = a_1$, the fixed points of the full 4-strategy game are the same those listed in Table I with two additional fixed points: $\mathbf{P}$ and

$$\mathbf{y}_3 \equiv \frac{2a_1 - 1}{Q'}\mathbf{R}_0 + \frac{a_2 + 1}{Q'}\mathbf{S} + \frac{a_1(a_2 - 1) + 1}{Q'}\mathbf{P}\ , \tag{10}$$

with $Q' \equiv (1 + a_1)(1 + a_2)$. One can verify that the conditions given in Table I for Nash equilibria or ESS hold for the 4-strategy game, that neither $\mathbf{P}$ nor $\mathbf{y}_3$ are Nash equilibria of Supplementary Eq. (9), and that there is no fixed point in the simplex interior.

For the reduced game on the $\mathbf{R}_0\mathbf{SP}$ face, $\mathbf{y}_3$ is a Nash equilibrium, but it is not an ESS (by the same reasoning used above to analyze $\mathbf{y}_2$). Trajectories in the interior of the $\mathbf{R}_0\mathbf{SP}$ face approach the heteroclinic cycle on the face boundary. Since $\mathbf{y}_3$ is a Nash equilibrium only in the reduced game, the eigenvector of the Jacobian in the direction transverse to the $\mathbf{R}_0\mathbf{SP}$ face has a positive eigenvalue, i.e. it is unstable.

In summary, the long-term outcome for the 4-strategy game given with Supplementary Eq. (9) with independent dispersion of phage is similar to that of the 3-strategy game given with Supplementary Eq. (8) of the main text. For $s > c$, there exist no stable fixed points and we find that trajectories in the simplex interior approach the heteroclinic cycle on the $\mathbf{R}_s\mathbf{R}_0\mathbf{S}$ face, while for $s < c$, $\mathbf{R}_s$ is the unique ESS of the system.
